# Impact of bovine lactoferrin supplementation and reduced iron in formula on infant oral microbiome: a randomized controlled trial

**DOI:** 10.1080/20002297.2025.2561212

**Published:** 2025-09-24

**Authors:** Cynthia Anticona, Anders Esberg, Staffan K. Berglund, Maria Björmsjö, Olle Hernell, Bo Lönnerdal, Ingegerd Johansson, Pernilla Lif Holgerson

**Affiliations:** a Department of Odontology, Umeå University, Umeå, Sweden; b Department of Odontology, Section of Cariology, Umeå University, Umeå, Sweden; c Department of Clinical Sciences, Pediatrics, Umeå University, Umeå, Sweden; d Department of Nutrition, University of California, Davis, CA, USA; e Department of Odontology, Section of Pediatric Dentistry, Umeå University, Umeå, Sweden

**Keywords:** Oral microbiota, infant formula, lactoferrin, iron supplementation, breast milk

## Abstract

**Introduction:**

Infant formulas with reduced iron levels and lactoferrin (Lf) supplementation might mimic the beneficial effects of breast milk on the oral microbiome. This study aimed to investigate the impact of a bovine Lf-supplemented and iron-reduced formula on the oral microbiota in infants at 4, 6 and 12 months.

**Methods:**

In a double-blind controlled trial, 6-week-old formula-fed infants were randomized to receive either a formula with reduced iron levels (2 mg/L) and Lf supplementation (1 g/L) (*n* = 72), the same formula without Lf (*n* = 72), or a standard formula (8 mg iron/L) (*n* = 36). A breast-fed reference group (*n* = 72) was also included. The oral microbiota was analyzed at 4 (*n* = 244), 6 (*n* = 216) and 12 (*n* = 229) months of age using the Oxford Nanopore Technology of the 16S rRNA gene annotation (*e*HOMD database).

**Results:**

Neither the within- or between-group diversities nor overall microbiota pattern assessment revealed any statistically significant differences in microbiota composition between the formula groups. However, single species were significantly associated with specific formula-fed groups. At 6 months, breast-fed infants exhibited significantly lower species richness and distinct microbiota composition compared to the formula-fed groups.

**Conclusions:**

The effects of reduced iron levels and lactoferrin supplementation of infant formula on the oral microbiome were inconclusive.

## Abstract (plain language summary)

The oral microbiome of breast-fed infants is more likely to carry health-promoting taxa, compared to their formula-fed counterparts.

Compared to breast milk, standard infant formula has a higher concentration of iron and lower level of lactoferrin. Hence, lowering the iron concentration and adding lactoferrin may help mimic the effects of breast milk on the gastrointestinal microbiome.

The effects of reduced iron levels and lactoferrin supplementation in infant formula on the oral microbiome yielded unclear and at its best, very modest effects from the iron reduction on the oral microbiome.

## Introduction

In addition to nutritional benefits, breastfeeding significantly reduces the risk of infections and supports critical physiological processes, including the maturation of the gastrointestinal (GI) microbiota in the child [1,2]. Hence, exclusive breastfeeding is recommended during the first six months of life, but despite its advantages, the global rates of exclusive breastfeeding remain low. Fewer than 50% of infants are reported to be exclusively breast-fed worldwide [3], with trends varying over time and location. For example, exclusive breastfeeding in infants up to four-month-old has decreased from 81% in 1997 to 61% in 2021 in Sweden, and partial breastfeeding risen from 12% in 1997 to 21% in 2021 [4]. To accommodate the nutritional needs of infants when breastfeeding is not exclusive, infant formulas are available. Although the composition of these formulas has been progressively refined to mimic breast milk more closely, significant differences in composition remain [5,6]. The LIME project [6,7], evaluating the effects of a low iron (Fe) and bovine lactoferrin (Lf) supplemented formula, is one example of an attempt to optimize infant formulas. In LIME, the iron concentration was reduced from 8 to 2 mg/L and 1 g/L of bovine Lf was added to mimic breast milk, which has Lf as a major protein and contains 0.1–1.6 mg Fe/L across different lactation stages [8].

Owing to the increased risk of iron deficiency in infancy, formula supplementation with iron is recommended. However, it has been suggested that iron concentrations in commercial formulas (8–14 mg/L) may be excessive with potential adverse effects, e.g. the promotion of opportunistic microorganisms [9]. Notably, 2 mg Fe/L in infant formula was found to be sufficient to meet the iron requirements of healthy infants with a low risk of deficiency [7,10], but this reduction is not applicable globally. Previous studies have shown that supplementing infant formula with bovine Lf, which shares 69% protein sequence identity with human Lf, affects iron status without adverse effects on growth or immunological health [6,7].

In addition to being a protein source, the intact Lf glycoprotein and its proteolytically released fragments/peptides affect the adhesion and metabolism of fungi and bacteria in the GI microbiomes [11,12]. The oral microbiome, the first part of the GI, matures during the first two years of life and is influenced by several factors, such as host genetics, antibiotic exposure and mode of birth and feeding [13,14]. Empirical studies indicate that breast-fed infants typically exhibit lower alpha diversity and are more likely to carry health-promoting taxa, such as probiotic lactobacilli, compared to their formula-fed counterparts [13,15]. Hence, lowering the iron concentration of infant formula and adding Lf may help mimic the effects of breast milk on the GI microbiome.

The primary aim of the present study, nested within the randomized controlled LIME trial [6,7], was to evaluate the impact of a bovine Lf-supplemented and iron-reduced formula introduced at 6 weeks of age on the oral microbiota composition at 4, 6 and 12 months of age. We hypothesized that infants receiving Lf-supplemented formula would exhibit an oral microbiota profile more akin to that of breast-fed infants, in contrast to those fed standard commercial formula. Additionally, the potential effect of formula with different iron concentrations on the oral microbiota was evaluated.

## Methods

### Study design and subjects

**Figure 1.**
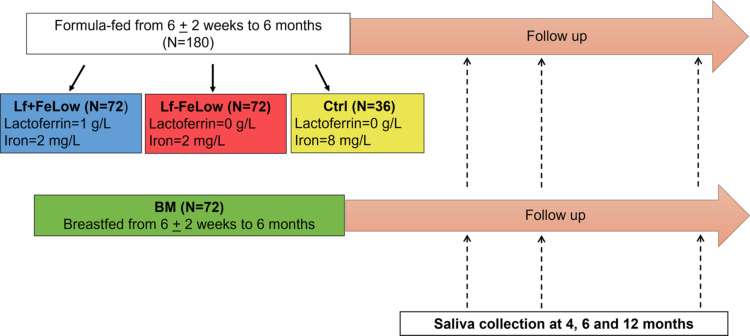
Study design. Lf + FeLow: experimental formula with low iron concentration (2 mg/L) and lactoferrin supplementation (1g/L); Lf − FeLow: experimental formula with low iron concentration (2 mg/L) and no lactoferrin supplementation; Ctrl: control formula (iron 8 mg/L); and BM: breast milk.

This study used baseline data from the LIME (Swedish acronym) project [6], a randomized, double-blind intervention study conducted in healthy Swedish infants from 6 ± 2 weeks to 6 months of age. The main aim of the LIME project was to assess the effect of adding 1 g/L of bovine lactoferrin and reducing the iron concentration in infant formula on infant's health and development. Infants were recruited and randomized to receive one of three formulas: (i) a control formula with an iron concentration adjusted from 12 to 8 mg/L to match Swedish standard formulas (Mead Johnson Nutrition, Evansville, IN, USA) (Ctrl, *n* = 36), (ii) an experimental formula based on the control formula but with less iron (2 mg/L) and fortified with 1.0 g/L bovine Lf (Hilmar Ingredients, Hilmar, CA, USA) with an iron saturation of 18% (Lf + FeLow, *n* = 72) or (iii) the same experimental formula without Lf supplementation (Lf − FeLow, *n* = 72) (Figure 1). A breast-fed reference group (BM, *n* = 72) was also recruited, and participants were matched with the other groups for age of birth. All groups were matched for gender. A detailed description of the recruitment and randomization is provided elsewhere [6,7]. The inclusion criteria were: birth weight 2500–4500 g, gestational age at birth ≥ 37 weeks, absence of chronic illness and neonatal diagnoses likely to affect any outcome, no previous blood transfusion or iron supplementation and exclusive formula feeding, and, for the reference group, exclusive breastfeeding at the inclusion and the intention to exclusively breastfeed until 6 months of age. The formula compositions are shown in Supplementary Table S1*.* Following national recommendations, all parents/caregivers were advised to avoid complementary foods before the infant was 4 months old and only give small amounts up to 6 months of age. Data on iron intake from complementary foods are provided in a previous publication [7]. Food and supplements with added pre or probiotics were not allowed.

As detailed previously [6,7], the sample size was based on pre-study power calculations to detect a significant difference in infant ferritin and cytokine levels at 6 months of age. Owing to the limited number of exclusively formula-fed infants eligible and to minimize the time duration of the study, the control group was smaller, while the groups receiving the two experimental formulas, Lf + FeLow and Lf − FeLow, were powered to explore the effects of lactoferrin with an effect size of 0.5 SD. To remain blinding, the Lf + FeLow and Lf − FeLow groups were both divided into two groups, for a total of 5 groups of 36 infants randomized to the 3 interventions. Data and biological samples collection were conducted at baseline (6 ± 2 weeks of age) and at the 4-, 6- and 12-months visits. Specific data and saliva samples were used in the present study (Figure 1).

### Saliva sampling

Saliva was collected using a specimen suction device (Unomedical, Malmö, Sweden), modified from Noakes et al., 2007 [16], and attached to a slight vacuum. The saliva was collected into sterile, ice-chilled test tubes, which were capped after collection and stored at −20 °C until being transferred to −80 °C within two days.

### DNA extraction

DNA was extracted from saliva samples and ultra-pure water (negative control) using the GenElute™ Bacterial Genomic DNA kit (Sigma–Aldrich, St. Louis, MO, USA) including lysozyme, mutanolysin, Proteinase K and RNase A enzymes as described previously [17]. DNA in the commercial ZymoBIOMICS Microbial Community DNA Standard, D6305 (NordicBiosite, Stockholm, Sweden) was used as the positive control. The quality of the extracted DNA was estimated using a NanoDrop 1000 Spectrophotometer and the quantity by the Qubit 4 Fluorometer (Thermo Fisher Scientific, Uppsala, Sweden).

### Bacteria 16S rRNA full gene amplicon sequencing

Full 16S rRNA gene sequencing was done using the Oxford Nanopore Technology. The v1 through v9 variable regions of 16S rDNA were amplified using 50 ng of DNA, KAPA 2x HiFi ready mix KAPA HiFi HotStart ReadyMix (2X) (New England Biolabs, Ipswich, MA, USA), and the primers 27F 5′-AGAGTTTGATCMTGGCTCAG-3′ and 1,492R 5′-CGGTTACCTTGTT ACGACTT-3′. This was performed on a MiniAmp™ Thermal Cycler (Thermo Fisher Scientific, Uppsala, Sweden) using the following program: 1 min denaturation at 98 °C, 35 cycles of 95 °C 20 s, 55 °C 15 s, 72 °C 1.5 min, and a final extension step of 1.5 min at 72 °C in 25 µL reactions. The creation of a single fragment of the expected 1,465 bp fragment was confirmed by separation on a 0.8% agarose gel in 0.5x Tris/Borate/EDTA buffer with SYBR™ Green (Fisher Scientific, Göteborg, Sweden). Sample amplicons were purified using the 0.8% AMPure XP Beads (Beckman Coulter, Brea, CA), washed twice in 80% ethanol and eluted in EB buffer (Fisher Scientific, Göteborg, Sweden), and quantified using the Qubit dsDNA HS Assay Kit and Qubit 4.0 Fluorometer (Thermo Fisher Scientific, Oregon, USA).

Library preparations were performed by barcoding amplicons using the Native Barcoding kit 96 V14 (SQK-NBD114.96) kit (Oxford Nanopore Technologies Nanopore, Oxford, UK). For this purpose, 200 fmol PCR products were end-repaired using NEBNext® Ultra™ II End Repair/dA-Tailing Module (NEB, E7546L) of which 10 fmol was used for ligation to unique barcodes for each sample using NEB Blunt/TA Ligase Master Mix (NEB, M0367). Barcoded samples were pooled and cleaned using 0.4x AMPure XP Beads (Beckman Coulter, Brea, CA). The purified and pooled samples were finally fused to the Native Adapter using T4 DNA Ligase (NEB, E6056) and finally cleaned with 0.4x AMPure XP Beads (Beckman Coulter, Brea, CA).

Libraries were quantified using a Qubit 4 fluorometer (Thermo Fisher Scientific, OR, USA). Sequencing of the Native Adapter barcoded pools was performed by loading 100 ng into a pre-primed R10.4.1 flow cell (Oxford Nanopore Technologies) and sequenced using a GridION nanopore sequencer (Oxford Nanopore Technologies) for 72 h. Base-calling of nanopore signals and demultiplexing was performed on the GriION using the MinKNOW (Nanopore, Oxford, UK), Dorado base callers Super accurate model and Porechop (version 0.2.4, https://github.com/rrwick/porechop) generating demultiplexed FastQ files, with a quality score (QC) score > 10 with a read length between 1,350 and 1,800 bp.

During the entire process, strict protocols were followed to mitigate external bacteria contamination, such as diligent cleaning of benches, sterile consumables, changes in tips between all steps, negative controls through all sample preparation steps from the DNA extraction until library preparation and with inclusion of the negative control in each 90-sample batch at random positions, and sensitivity classifying of the negative controls to a database including non-oral bacteria. None of the runs showed signs of contamination.

#### Sequence processing

The open-source Emu pipeline (https://github.com/treangenlab/emu; Curry et al., 2022) was used for sequence processing with the abundance filter set to > 0.0001 [--min-abundance 0.0001]. Samples with < 10,100 reads were excluded. The sequences from the retained samples were classified against the extended Human Oral Microbiome Database RefSeq (*e*HOMD 16S rRNA RefSeq Version 15.23) covering 1,015 16S rRNA sequences from 774 bacterial species in the oral cavity, pharynx, nasal passages, sinuses and esophagus [18]. Taxa with a minimum of two reads in at least two samples were retained.

### Data analyses

Descriptive data included participants' gestational age at birth, birth weight, as well as weight and length assessed at the 4-, 6- and 12-months visits are presented as means with 95% confidence interval (CI) and differences were tested with analysis of variance (ANOVA). Categorical variables, including sex and child delivery method, are presented as proportions and the Chi-square test was used to test differences between the groups. The prevalence of bacterial species (proportion of participants for whom a species was present) and relative abundances were calculated and used in descriptive and multivariate analyses were detected. STATA, version 17 (StataCorp LLC, College Station, TX, USA) was used for descriptive analyses and univariate testing of differences.

Species found in all (100%) samples that were shared between groups were identified in a Venn diagram using Interactive Venn (​​​​​​http://www.interactivenn.net/) [19].

Microbiota diversity was assessed as alpha diversity using three indices: the observed number of species, Shannon diversity and Pielou evenness index. Beta diversity was assessed using Bray–Curtis dissimilarity index. Statistical comparisons included *p*-values and effect size estimates.

Diversity assessments used microbiota counts rarified at 10,100 reads using the Vegan package in the Microbiome process for R studio.

Principal component analysis (PCA) was used to search for clustering across participants, and multivariate orthogonal partial least square regression (OPLS/OPLS-DA with feeding allocation as the dependent variable and species abundancies as independent variables) was used for targeted analyses using SIMCA P + (version 18.0, Sartorius Stedim Data Analytics AB, Umeå|Malmö, Sweden). The variables were scaled to unit variance and the models were cross-validated by systematic testing after the exclusion of every 7^th^ participant. Hence, model explanatory (*R*^2^) and predictive (*Q*^2^) values were obtained. The results are displayed in score and loading plots where each symbol represents an observation, and variable loadings identify the impact of each variable. The loadings are expressed as variable importance in projection (VIP) values and OPLS correlation coefficients *p*(corr). Loadings that do not include 0 (zero) in the 95% CI are statistically significant. Volcano plots based on VIP-values and *p*(corr) values were used to identify the most influential species in the models. The association between species abundance and the feeding group was then evaluated in linear regressions to allow for the inclusion of covariates (here, the mode of delivery and for evaluation of effects of iron level and Lf supplementation). Sensitivity analyses, including sex and birth weight were run for all outcomes but did not affect the results.

## Results

### Study group characteristics

In total, 689 saliva swab samples were collected and sequenced from the infants (244 at 4 months, 216 at 6 months and 229 at 12 months). Of these, 668 samples had ≥ 10,100 reads and were retained (Table 1).

**Table 1.** Background characteristics of the infants by feeding group and age.

	Study groups				
	Lf + FeLow	Lf − FeLow	Ctrl	BM	*p*-Value
**4 months of age**					
Saliva samples, *n*	**70**	**71**	**35**	**68**	–
Microbiota evaluation, *n*	**64**	**65**	**34**	**65**	–
Girls, *n* (%)	33 (50.6)	33 (50.7)	17 (50.0)	32 (49.2)	0.995
Gestational age at birth (weeks), mean (95%CI)	39.7 (39.5, 40.1)	39.8 (39.5, 40.1)	39.5 (39.1, 40.0)	39.7 (39.5, 40.0)	0.567
Birth weight, kg, mean (95%CI)	3.6 (3.5, 3.7)	3.5 (3.4, 3.6)	3.5 (3.4, 3.6)	3.5 (3.4, 3.6)	0.394
C-section, *n* (%)	19 (29.7)	15 (23.1)	8 (23.5)	9 (13.8)	0.192
Weight, kg, mean (95%CI)	6.9 (6.8, 7.2)	6.9 (6.8, 7.1)	7.0 (6.7, 7.3)	6.8 (6.5, 6.9)	0.222
Length, kg, mean (95%CI)	63.4 (62.9, 63.8)	63.2 (62.7, 63.6)	63.1 (62.3, 63.9)	63.0 (62.5, 63.6)	0.795
**6 months of age**					
Saliva samples, *n*	**63**	**65**	**27**	**61**	–
Microbiota evaluation, *n*	**63**	**65**	**26**	**60**	–
Girls, *n* (%)	33 (52.4)	33 (50.7)	10 (38.5)	33 (55.0)	0.558
Gestational age at birth (weeks), mean (95%CI)	39.5 (39.1, 39.8)	39.8 (39.5, 40.1)	39.4 (38.9, 39.8)	39.7 (39.4, 39.9)	0.264
Birth weight, kg, mean (95%CI)	3.6 (3.5, 3.7)	3.5 (3.4, 3.6)	3.6 (3.4, 3.7)	3.5 (3.5, 3.6)	0.773
C-section, *n* (%)	19 (30.2)	14 (21.5)	7 (26.9)	10 (16.7)	0.330
Weight, kg, mean (95%CI)	8.1 (7.9, 8.3)	8.2 (7.9, 8.4)	8.4 (8.0, 8.7)	7.7 (7.5, 7.9)	0.007
Length, kg, mean (95%CI)	67.5 (66.9, 68.0)	67.2 (66.7, 67.7)	67.5 (66.7, 68.4)	66.1 (65.5, 66.7)	0.001
**12 months of age**					
Saliva samples, *n*	**68**	**67**	**31**	**63**	–
Microbiota evaluation, *n*	**67**	**67**	**30**	**62**	–
Girls, *n* (%)	34 (50.8)	33 (49.3)	14 (46.7)	33 (53.2)	0.940
Gestational age at birth (weeks), mean (95%CI)	39.6 (39.3, 39.9)	39.8 (39.5, 40.0)	39.5 (39.1, 39.9)	39.9 (39.6, 40.1)	0.328
Birth weight, kg, mean (95%CI)	3.6 (3.5, 3.7)	3.5 (3.4, 3.6)	3.5 (3.4, 3.7)	3.5 (3.5, 3.6)	0.768
C-section, *n* (%)	19 (28.4)	14 (20.9)	6 (20.0)	9 (14.5)	0.291
Weight, kg, mean (95%CI)	10.2 (9.9, 10.4)	10.2 (9.9, 10.5)	10.3 (9.8, 10.7)	9.8 (9.5, 10.0)	0.124
Length, kg, mean (95%CI)	75.8 (75.3, 76.4)	75.8 (75.3, 76.4)	75.7 (74.7, 76.7)	74.9 (74.2, 75.6)	0.115

*p*-Values for differences among the test and control groups were calculated using ANOVA test and Chi^2^ test for proportions. Lf + FeLow: experimental formula with low iron concentration (2 mg/L) and lactoferrin supplementation; Lf − FeLow: experimental formula with low iron concentration (2 mg/L) and no lactoferrin supplementation. Ctrl: control formula. BM: breast milk.

In addition to all formula-fed groups exhibiting higher average weights and lengths at 6 months compared to their breast-fed counterparts, no significant differences were observed in other characteristics, such as sex, gestational age at birth and type of delivery across the feeding groups at various screening ages (Table 1).

### Overall sequencing results

Among the 668 retained samples, 350 phylotypes were identified. They consisted of 234 named species (19 with strain variations) and 116 unnamed phylotypes across 98 genera and 12 phyla when each phylotype was represented by at least two sequences in at least two samples. At 4 months of age, 254 phylotypes were detected, which were similar at 6 months (255 phylotypes), but rose to 313 phylotypes by 12 months. The detection prevalence rates and relative abundances of these phylotypes, stratified by feeding group and age, are detailed in Supplementary Table S2.

 At 4 months, the core microbiome (present in all infants) consisted of nine phylotypes in the *Alloprevotella, Gemella, Rothia, Streptococcus and Veillonella* genera. The core expanded to 17 phylotypes by 6 months, and to 33 phylotypes across five additional genera by 12 months (Supplementary Table S3). Approximately 40% of the phylotypes detected at 4 and 6 months were shared across all the feeding groups, a proportion that increased to 65% by 12 months. The distribution of shared species among the four feeding groups are illustrated in Venn diagrams in Supplementary Figure S1.

### Multivariate search for microbiota clustering across feeding groups

**Figure 2.**
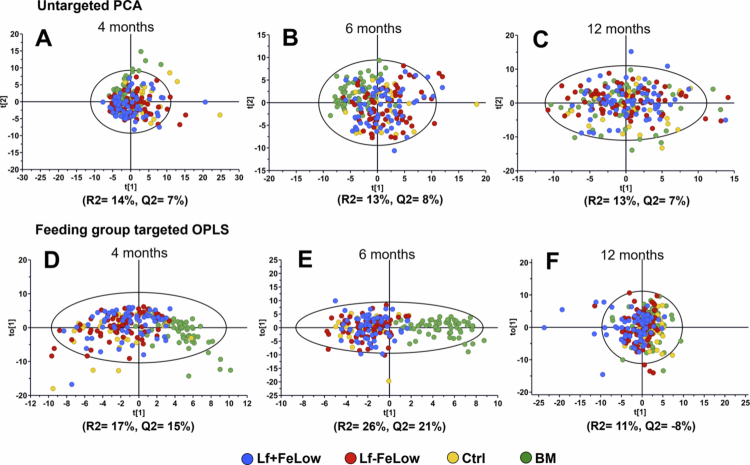
Loading plots from untargeted PCA (upper panel) and feeding group targeted OPLS (lower panel). Data are presented for 4 months (A, D), 6 months (B, E) and 12 months (C, F) old infants and species relative abundancies are shown as an independent matrix. Each dot represents an infant and coloring the four feeding groups, i.e. experimental formula with low iron concentration and lactoferrin supplementation (Lf + FeLow); experimental formula with low iron concentration and no lactoferrin supplementation (Lf − FeLow), control formula (Ctrl) and breast milk (BM).

Potential clustering of bacterial species corresponding to the four feeding groups was searched by untargeted PCA across age strata. The PCA models exhibited low explanatory power, with *R*^2^ values of 13 and 14%, and although the breast milk (BM) group tended to stand out at 6 months, no PCA model reached statistical significance (Figure 2A–C, upper panel). To follow up on this, feeding group targeted OPLS was performed using the same x-matrix.

The OPLS models were slightly stronger and statistically significant for 4- and 6-months old infants (*R*^2^ = 17, *Q*^2^ = 15) % and (*R*^2^ = 26, *Q*^2^ = 21) %, respectively (Figure 2D–F, lower panel). Generally, infants in formula-fed groups were interspersed in the OPLS score plots at all ages but with a tendency to distinction at 6 months of age, whereas breast-fed infants were clustered separately at 4 and 6 months of age (Figure 2E). Notably, separation of breast-fed versus formula-fed infants was not observed when the infants were 12 months old.

### Exploring microbiota patterns across feeding modes at 6 months of age

Given the strongest feeding effects at 6 months, according to the OPLS regression, we continued with a detailed exploration of this age group. At this age, rarefaction curves of the number of observed species and the Shannon diversity index (alpha diversity) revealed significantly greater diversity in all formula-fed groups compared to their breastfed counterparts (*p *< 0.001) (Supplementary Figure S2A and S2B). Although less evident, and only statistically significant for the Lf − FeLow vs. Ctrl group (*p* = 0.046), a higher Shannon index among infants fed the low-iron formulas suggested that iron content affected the within group diversity (Supplementary Figure S2B).

The beta diversity, assessed as the Bray‒Curtis dissimilarity index and displayed in a PCoA plot with associated boxplots for the two components, demonstrated less difference across formula-fed infants than between formula-fed and breast-fed infants (*p *< 0.001, Supplementary Figure S2C). The pattern was consistent for both components 1 and 2. A series of pairwise comparisons of the Bray‒Curtis dissimilarity index displayed, besides the consistent distinction of BM and each formula type, a significant difference between infants fed the control formula and those with low iron and lactoferrin supplementation (Ctrl vs. Lf + FeLow, *p* = 0.048), and a near-significant difference between infants fed low-iron formula with and without Lf supplementation (Lf + FeLow vs. Lf − FeLow, *p* = 0.069) (Supplementary Figure S2D). However, effect size estimates from ANOVA suggested that the observed differences may not be meaningful (Eta-squared = 0.428, 95% CI: 0.376–0.466).

**Figure 3.**
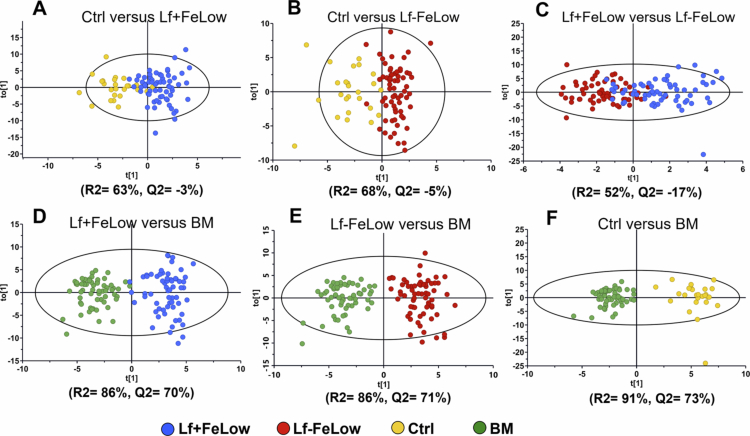
Score plots from the formula group targeted OPLS-DA with bacterial species at 6 months as the x-matrix. The dots represent individual infants in systematic comparisons of experimental formulas (Lf + FeLow and Lf − FeLow) when comparing control formula (Ctrl) versus a formula with low iron concentration and lactoferrin supplementation (Lf + FeLow) (A); Ctrl versus a formula with low iron concentration and no lactoferrin supplementation (Lf − FeLow) (B); Lf + FeLow versus Lf − FeLow (C); Lf + FeLow versus BM (D), Lf − FeLow versus BM (E) and Ctrl versus BM (F).

As a next step, multivariate formula group targeted OPLS-DA models, i.e. pairwise comparisons employing species relative abundancies, were run to follow up on the Bray‒Curtis dissimilarity finding. The obtained models had high explanatory (*R*^2^) values (52–91%) but, apart from comparisons with breast milk (Figure 3D, F), the predictive values were negative (Figure 3A–C) indicating that the latter differences using the whole microbiota profile were unstable. A weakness of PLS/OPLS is that confounders may be included in the model but not adjusted for, meaning that single influential species may remain undetected or be overestimated. Therefore, we continued with multiple regressions where covariates were adjusted for.

**Figure 4.**
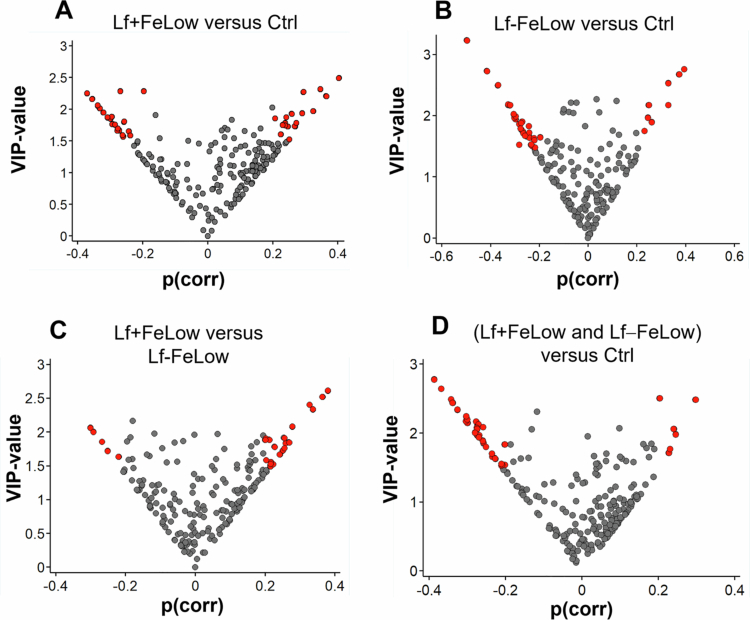
Volcano plots of OPLS-DA-derived correlation coefficients versus VIP-values when infants were 6 months old. The comparisons are: the experimental formula with low iron concentration and lactoferrin supplementation (Lf + FeLow) versus the control formula (Ctrl) (A); the experimental formula with low iron concentration and no lactoferrin supplementation (Lf − FeLow) versus the Ctrl formula (B); the two experimental formulas (Lf + FeLow versus Lf − FeLow) (D); and the formulas with low Fe (Lf + FeLow and Lf − FeLow) versus the Ctrl formula (E). The red dots indicate the most influential bacterial species, i.e. OPLS-DA correlation coefficient *p*(corr) > 0.2 and VIP-value > 1.5.

To identify candidate phylotypes, we identified the species that were most influential in the OPLS-DA models from Volcano plots showing OPLS-DA correlation coefficients against VIP-values (Figure 4A–D). Species with an OPLS-DA correlation coefficient *p*(corr) > 0.2 and a VIP-value > 1.5 were selected for linear regression analysis where mode of birth and, when comparing iron levels, also Lf supplementation, were included as covariates.

The phylotypes selected for the comparisons shown in Figure 4A–C consisted of 37 phylotypes for Lf + FeLow versus Ctrl (Figure 4A), 34 for Lf − FeLow versus Ctrl (Figure 4B) and 25 for Lf + FeLow versus Lf − FeLow (Figure 4C). Some phylotypes remained significant after adjusting for the mode of delivery in linear regression (Supplementary Table S4).

In the comparison of Lf + FeLow versus Ctrl, four of the eight significant phylotypes were associated with Lf + FeLow formula (most influential: *Alloscardovia omnicolens*, *p* = 0.007), and four were associated with the Ctrl formula (most influential: *Streptococcus infantis*, *p* = 0.012). For the Lf − FeLow versus Ctrl comparison, eight of the 12 significant phylotypes were associated with the Lf − FeLow formula (most influential: *Alloscardovia omnicolens*, *p* = 0.007) and four with the Ctrl formula (most influential: *Granulicatella adiacens*, *p* = 0.002). In the Lf + FeLow versus Lf − FeLow comparison, seven of the eight significant phylotypes were associated with the Lf + FeLow formula (most influential: *Anaerolinea thermophila*, *p* = 0.009) and one with the Lf − FeLow formula (most influential: *Prevotella melaninogenica*, *p* = 0.024).

### Exploring microbiota patterns across formula iron levels at 6 months of age

**Figure 5.**
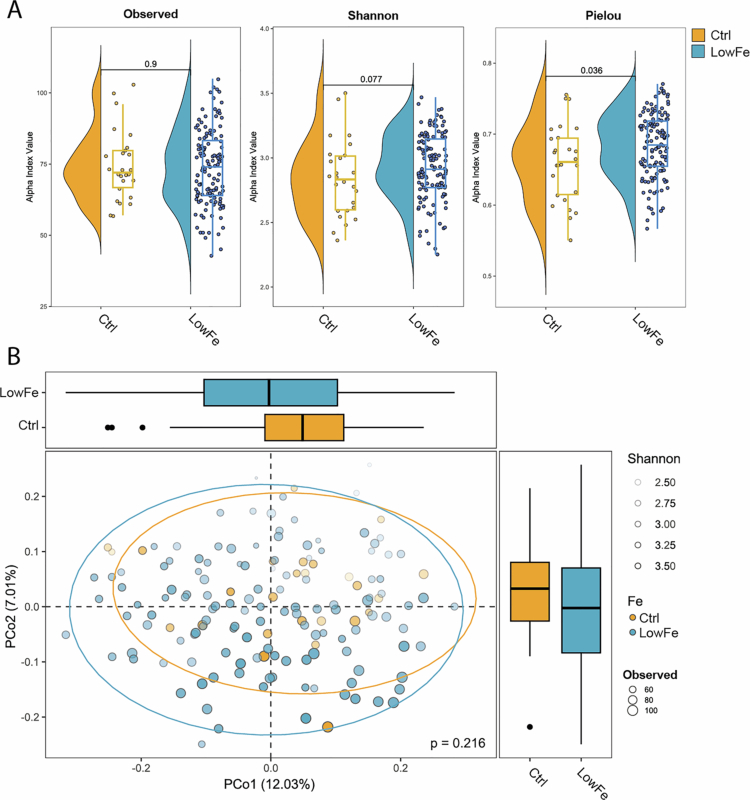
Oral microbiota diversity between infants fed low iron formulas versus the control formula at 6 months of age. The low iron (LowFe) formulas including Lf − FeLow and Lf + FeLow with 2 mg/L Fe and the control formula (Ctrl) with 8 mg/L Fe. Observed number of species, Shannon diversity index and Pielou evenness index (A) and PCoA plot illustrating the Bray‒Curtis dissimilarity index in the infants' oral microbiota (B). The color reveals feeding group identity and the box plots median (25, 75%) limits values for components 1 and 2.

To further investigate the potential impact of iron concentration on the oral microbiota, as suggested in Supplementary Figure S2, we compared the microbiota composition between infants who received either of the two low iron (LowFe) formulas (Lf + FeLow or Lf − FeLow), each containing 2 mg/L and those receiving the control formula (Ctrl), containing 8 mg/L in diversity and linear regression analysis. The species richness did not differ significantly between the LowFe and control groups, but the species evenness (Pielou index, *p* = 0.036) was greater among infants fed the Low-Fe formulas (Figure 5A). To account for the potential effect of Lf, we performed a direct comparison between the control and Lf − FeLow groups, which revealed a similar trend (Pielou index, *p* = 0.034; Supplementary Figure S2E). There was no systematic difference in the microbiota profiles between the two iron concentration groups (Figure 5B). Among the 35 phylotypes selected for comparing the control versus the Low-Fe groups (Figure 4D), 11 remained significant when the mode of delivery and the presence or absence of Lf were included as covariates. All were associated with being fed a low iron formula (most influential *Alloscardovia omnicolens*, *p* = < 0.001) (Supplementary Table S4).

## Discussion

This study examined the oral microbiota composition of infants fed two experimental iron-reduced formulas, with and without bovine Lf supplementation, and compared it to that of infants fed standard formula or breast milk. The initial assessment of the species richness suggested a potential effect of the iron level on the alpha diversity and of Lf on the beta diversity, though this could not be confirmed when comparing the overall species composition in the OPLS-DA models at six months of age. However, single species driving the difference between the formula-fed groups were identified when adjusting for potential confounders in separate multiple linear regressions. In contrast, a clear distinction in the oral microbiota between children receiving formula (regardless of type) and breast-fed children was confirmed as long as exclusive breastfeeding continued.

The greater alpha diversity (both from the observed number of species and Shannon diversity) and higher Bray‒Curtis dissimilarity, i.e. less close clustering of infants, in formula-fed compared to breast-fed infants, is consistent with previous research [20,13], suggesting that the more homogeneous composition of the oral microbiome in breast-fed infants may result from the presence of probiotic *Lactobacilli* and *Streptococci* [21] and a panel of bioactive components in breast milk [13]. Among formula-fed infants, the panel of detected species was similar, but compared to those fed the control formula, the species evenness (Pielou index) was greater in infants fed formulas with lower iron concentrations. This finding may suggest that the higher iron concentration in the control formula compared to the experimental formulas led to the enrichment of certain species and a less uniform distribution of detected species. Many bacterial species need iron in the growth medium, and that high iron intake or supplementation with iron in starving infants may promote the enrichment of selected species [22]. While the alpha diversity seemed more affected by the iron level, the beta diversity differed according to the presence or absence of Lf. A clear distinction of these effects was, however, limited by the design of this study. Nevertheless, these initial findings were not supported by further OPLS-DA regression analysis, which showed separation between all the formula groups at 6 months of age, but did not survive cross-validation and thus lacked power to predict group allocation from the microbiota profile.

Taken together, the modest impact of Lf supplementation and a reduction of iron concentration on the oral microbiome aligns with the findings of the clinical trial in which this study was nested, reporting no evident or lasting effect on any of the investigated outcomes, including the inflammatory response, morbidity, growth and iron status [6,7]. The only significant clinical group differences observed were as expected between formula-fed infants and breast-fed infants (not included in the randomization). The authors' potential explanations were the undefined dose‒response effects of Lf and the healthy status of the participants, which contrasts with previous study populations that reported higher morbidity and mortality rates [6,7]. Furthermore, our findings support most recent clinical research examining the effects of lactoferrin oral administration on the infant gut microbiota [23,24]. One study in children aged 12–18 months suggested that lactoferrin oral treatment did not affect either gut microbiome diversity or composition over time [23]. Another study in preterm infants reported minimal impacts of lactoferrin on the gut microbiome or metabolome [24]. Nevertheless, *in vitro* studies have reported impacts on bacteria and endothelial function. The lack of consistent effects suggests that compared with other components of breast milk, e.g. human milk oligosaccharides, the effect of Lf on the gut microbiome may be limited [25].

In the present study, we hypothesized that the antibacterial activity of Lf would positively affect the oral microbiome of the infants fed the Lf-supplemented formula, resulting in an oral microbiome more similar to that of breast-fed infants rather than to those fed standard formula. It may be speculated that factors related to the dosage of Lf [26] explain the lack of such results. The optimal dosage at which Lf can exert a beneficial effect is still unknown. The maximum amount of added bovine Lf in infant formula in Europe is regulated at 1 g/L [27], which is what we applied in this study. Mature human milk contains 1−3 g/L Lf, and it is conceivable that the dosage of bovine Lf was insufficient to affect the outcomes studied. Another explanation might be the structural differences between human and bovine Lf (30% difference in amino acid sequence), as they imply differences in antimicrobial and antibiofilm effects, among other functional properties [28]. Other factors that could influence the results include the preservation of the Lf bioactivity during infant formula processing [29] and interactions with other nutrients in the composition of the infant formula [26,30].

The specific effect of lowering the iron concentration of infant formula on the composition of the oral microbiome was not the focus of this study. However, it was expected that the oral microbiome of the infants fed the reduced-iron formula with Lf supplementation would more closely resemble that of breast-fed infants compared to those fed the other two formulas, at least after several months of exposure, allowing for microbiota adaption. Additionally, the supplementation of formula with bovine Lf could emulate some of the antimicrobial mechanisms of human Lf in breast milk. Owing to its high affinity for ferric iron, Lf deprives microbes from free iron necessary for their growth, inhibiting their proliferation in the intestine (and possibly in the oral cavity) [31], whereas a relatively high content of Fe (without Lf addition) promotes growth. Nevertheless, our findings show that, given the levels used in this study, the microbiome composition was largely similar across formula-fed groups throughout the test period. In addition to iron and lactoferrin levels, other differences between breast-fed and formula-fed infants not addressed in the LIME intervention may explain these findings.

This study has notable strengths. First, the randomized design of the clinical trial with a high compliance to the intervention. Second, the full-length 16S rRNA gene was used for taxonomic classification, and that sequences could be taxonomically defined against a curated 16S rDNA database specifically built for oral bacteria [18]. However, the study also has limitations. One major limitation was the absence of an intervention group fed a formula containing 8 mg/L Fe with lactoferrin supplementation, as this would provide the opportunity to assess the potential intersecting effects of iron and Lf on the oral microbiome. Furthermore, the trial was well powered concerning primary outcomes, which were iron status and immunological effects. However, the power related to other outcomes, including that investigated in this study, was not addressed. Additionally, saliva sample collection was not adjusted to time from the last feeding because of the young age of the participants and their frequent feeding patterns. This may have introduced potential confounding factors. The use of low-biomass samples also presents challenges, as it increases the risk of contamination and may introduce confounding factors in microbiota analyses. Furthermore, reliance on the eHOMD database, while appropriate for oral taxa, may limit the detection of potential contaminants or non-oral taxa, as it excludes organisms not typically associated with the human microbiome. Finally, the homogeneity of the study population formed by healthy term infants living in a good socioeconomic situation limits the generalizability of these findings to other populations.

## Conclusions

In summary, the effects of reduced iron levels and lactoferrin supplementation in infant formula on the oral microbiome yielded unclear and at its best, very modest effects from the iron reduction on the oral microbiome. While reduced iron levels appeared to influence species evenness and lactoferrin beta-diversity, the overall species composition did not significantly differ among the formula-fed groups at six months of age.

## Supplementary Material

Supplementary material Supplementary material

Supplementary material Figures S1 and S2 Supplementary material Figures S1 and S2

## Data Availability

All relevant data are included in the paper or available through Supplementary information files. Sequence data can be found here: https://doi.org/10.6084/m9.figshare.30134767.v1.
