# Supplementary material for: Impact of bovine lactoferrin supplementation and reduced iron in formula on infant oral microbiome: a randomized controlled trial
Source: J Oral Microbiol. 2025 Sep 24;17(1):2561212. doi: 10.1080/20002297.2025.2561212 (PMC12466180; doi:10.1080/20002297.2025.2561212)

Figure S1. Venn diagram illustrating taxa overlaps between feeding modes at 4 months (A), 6 months (B) and 12 months (C) and an attached list of the taxa identified in 100% of the samples at each age group. Experimental formula with low iron concentration (2 mg/L) and lactoferrin supplementation (Lf+FeLow); experimental formula with low iron concentration (2 mg/L) and no lactoferrin supplementation (Lf-FeLow); control formula (Ctrl); breast milk (BM).


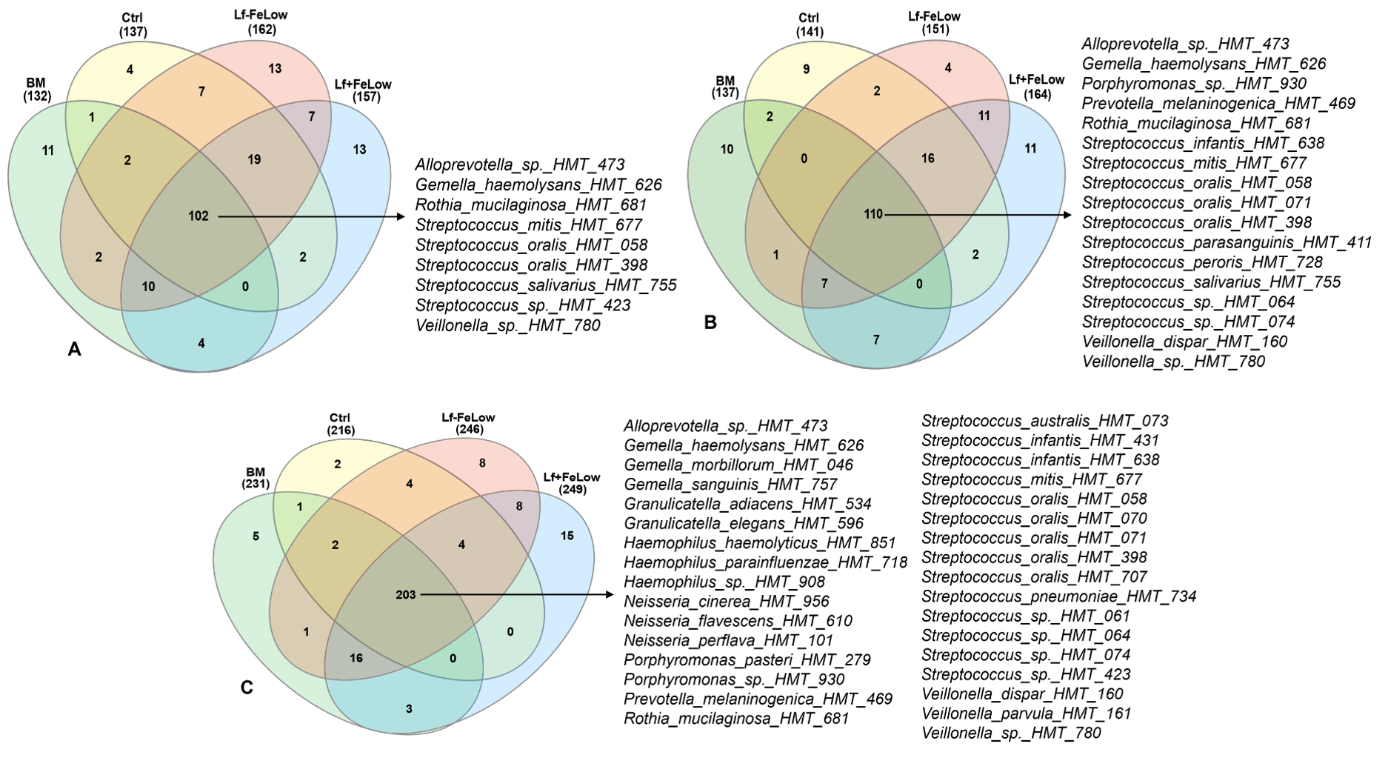


Figure S2. Oral microbiota diversity between infants fed the experimental formulas (Lf-FeLow, Lf+FeLow), the control formula (Ctrl) or breast milk (BM) up to 6 months of age. Rarefaction curves showing observed number of species (A) and Shannon diversity index by increasing sequencing depth (B). PCoA plot illustrating Bray-Curtis dissimilarity in the infants’ oral microbiota. Coloring reveals feeding group identity and the box plots median (25, 75% limits values for component 1 and component 2) (C). Box plots illustrating the Bray-Curtis dissimilarity per sample within each group and against the rest, i.e., BM versus other groups, Ctrl formula versus experimental formulas, and experimental formula with Lf supplementation (Lf+FeLow) versus that without (Lf-FeLow) (D). Alpha diversity comparison of the Controls versus Lf-FeLow (E).


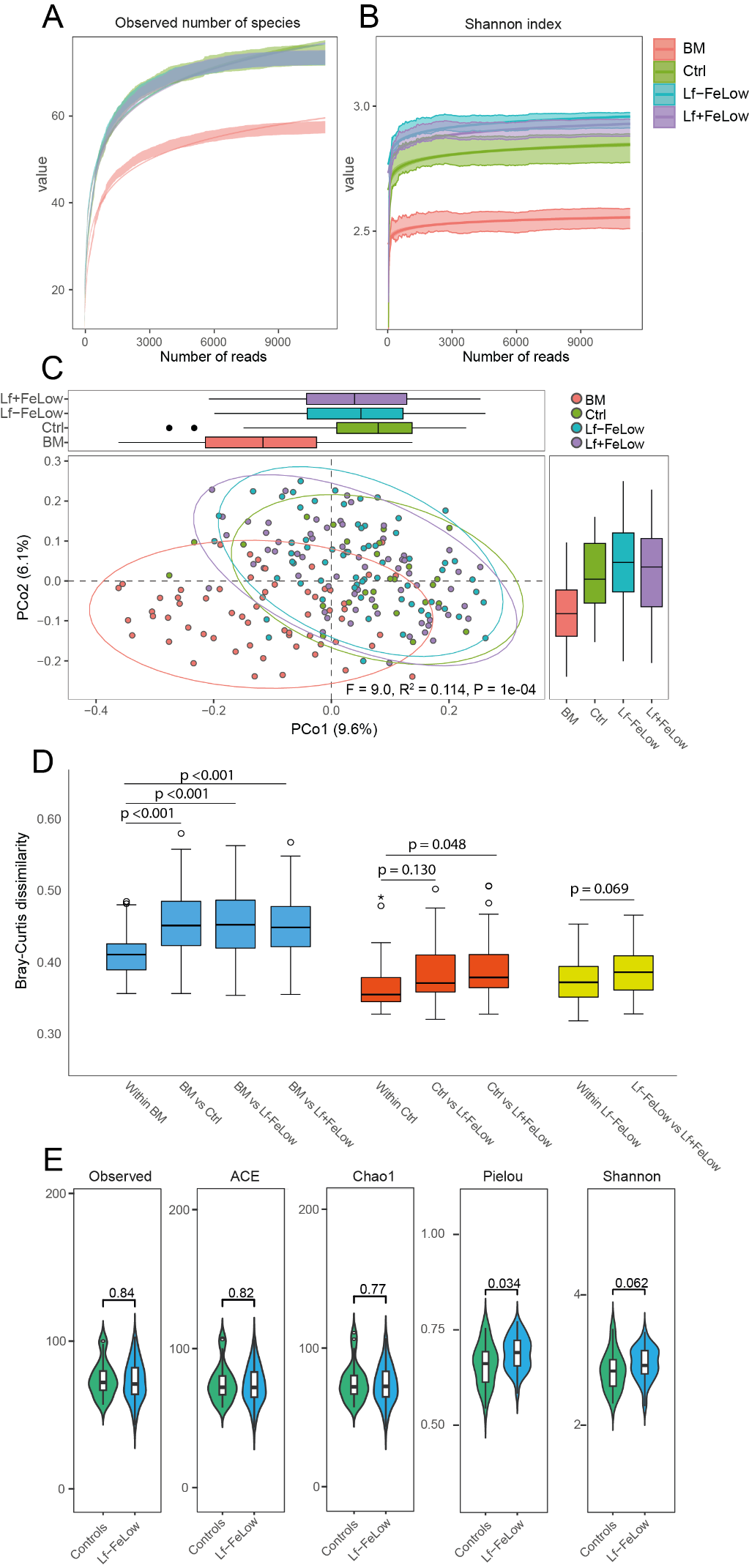

Supplement: Supplementary material Figures S1 and S2 [file ZJOM_A_2561212_SM0182.docx]
